# Influence of the Peek Abutments on Mechanical Behavior of the Internal Connections Single Implant

**DOI:** 10.3390/ma15228133

**Published:** 2022-11-16

**Authors:** Jefferson David Melo de Matos, Guilherme da Rocha Scalzer Lopes, Daher Antonio Queiroz, André Luiz Jesus Pereira, Mário Alexandre Coelho Sinhoreti, Nathália de Carvalho Ramos, Vinicius Lino, Flavio Rosa de Oliveira, Alexandre Luiz Souto Borges, Marco Antonio Bottino

**Affiliations:** 1 Department of Restorative Dental Sciences, Center for Dental Biomaterials, University of Florida (UF Health), Gainesville, FL 32601, USA; 2 Department of Biomaterials, Dental Materials, and Prosthodontics, São Paulo State University (Unesp), Institute of Science and Technology, São José dos Campos 12228-900, SP, Brazil; 3 Department of Restorative Dentistry & Prosthodontics, School of Dentistry, The University of Texas Health Science Center at Houston (UTHealth), Houston, TX 77054, USA; 4 Plasmas and Processes Laboratory, Physics Departament, Aeronautics Technological Institute (ITA), 50 Marechal Eduardo Gomes Square, São José dos Campos 12228-900, SP, Brazil; 5 Department of Restorative Dentistry, Dental Materials Division, Piracicaba Dental School (FOP-UNICAMP), Piracicaba 13416-000, SP, Brazil; 6 Department of Dentistry, Universidade São Francisco (USF), Bragança Paulista 12916-900, SP, Brazil; 7 Department of Dentistry, Postgraduate Program in Dentistry, University of Taubaté (UNITAU), Taubaté 12080-000, SP, Brazil

**Keywords:** bioengineering, dental abutments, dental implants, dental materials, polyetheretherketone

## Abstract

The present study aimed to evaluate the biomechanical behavior of PEEK abutments with different heights on single titanium implants. To investigate the implant surface, different tests (scanning electron microscopy, energy-dispersive X-ray, and X-ray diffraction) were adopted. Herein, 20 implants received the 4.5 × 4.0 mm PEEK short abutment (SA) and 20 received the 4.5 × 5.5 mm PEEK long abutment (LA). The abutments were installed using dual-cure resin cement. To determine the fatigue test, two specimens from each group were submitted to the single load fracture test. For this, the samples were submitted to a compressive load of (0.5 mm/min; 30°) in a universal testing machine. For the fatigue test, the samples received 2,000,000 cycles (2 Hz; 30°). The number of cycles and the load test was analyzed by the reliability software SPSS statistics using Kaplan-Meier and Mantel-Cox tests (log-rank) (*p* < 0.05). The maximum load showed no statistically significant differences (*p* = 0.189) for the SA group (64.1 kgf) and the LA group (56.5 kgf). The study groups were statistically different regarding the number of cycles (*p* = 0.022) and fracture strength (*p* = 0.001). PEEK abutments can be indicated with caution for implant-supported rehabilitation and may be suitable as temporary rehabilitation.

## 1. Introduction

The use of dental implants for oral rehabilitation has become a common condition in the clinical routine since it improves individuals’ quality of life [1]. In this sense, the main type of internal connection used in oral rehabilitation is the morse taper system, since it is an implant system with a mechanically precise internal connection with the prosthetic component, in which the abutment has a narrower shape in its base, which is coupled with the morse fitting/connection inside the implant, with intimate contact. This intimate contact is defined by some authors as a cold soldering system or fitting through intimate mechanical coupling without heat [2,3]. In addition, most systems on the market have threads and/or hexagons in the lower portion of the column to guide its placement. The development of this type of connection aims to direct the physiological loads to the apical region of the implant body, directing them to the medullary bone. In this sense, a space-free connection is sought between the interface region of the implant platform and the prosthetic component [1,2,3,4].

The macrogeometry design of morse tapered implant screws is characterized by the internal walls of the implant and the external walls of the abutment fabricated with an 8° taper. During the threading of the abutment into the implant body, there is intimate contact between the two components, creating frictional locking [1]. Friction between two slightly divergent surfaces, combined with a pressure created by the insertion force, secures the male cone to the female cone. This design promotes significant retention and strength under lateral loads, creating frictional adaptation to the internal anchorage or implant body, allowing for an extended duration of the function [2].

The success due to the implant in the biomechanical control of alveolar crest treatment is crucial for long-term bone loading conditions of the rehabilitated, since, in the osseointegration process, it is not crucial, followed by bone resorption of the alveolar crest [3]. Studies indicate that the masticatory loads on supported rehabilitations can generate occlusal overload, considered one of the main factors of mechanical and biological failures [3]. The fillers are not manufactured with quality and there is a consensus that the location is original, with a magnitude in all components of the prosthesis/produced [5,6,7]. The application of functional loading induces stress and tension in the bone/implant complex and affects peri-implant bone remodeling [8,9].

Some implant failures may be related to unfavorable stress magnitudes [10,11,12,13]. When pathological overload occurs, stress gradients exceed physiological bone tolerance and cause microfractures at the bone/implant interface [14]. Occlusal overload results in increased bone resorption around the implant and decreased percentage of mineralized bone tissue [15], showing that a remodeling process occurs when a bone is subjected to stress [16,17].

The rehabilitation of implant-supported prostheses allows the use of numerous components with different retention systems. However, the versatility of CAD/CAM abutments and their technological advantages have been part of the prosthetic solution of numerous companies, since these abutments have brought the advantage of using cement-retained prostheses, by filling the space between the crown and the abutment, in addition to allowing access to the fixation structure in the cortical bone [5]. This technology allows the prosthetic elaboration of a structure between the abutment and crown and even the cementation of the ceramic crown directly on the implant, with direct wear of the abutment simulating the condition of tooth preparation [6,7].

Therefore, it is essential to understand how masticatory loads can influence the biomechanical behavior of rehabilitation with osseointegrated implants using this rehabilitated system, since similar prosthetic abutments may present different behaviors for the same load [8]. Even with the availability of CAD/CAM abutments with different heights among different implant companies, there are still no studies on peripheral stresses and strains, namely, little information is available regarding the influence of this magnitude on the biomechanical behavior of these restorations [9].

Therefore, studies have been carried out to improve the understanding of the influence of different materials and heights on the mechanical behavior of implant-support rehabilitation [10,11]. When different methodologies are used in the same investigation, they allow for analysis with fewer views, in addition to allowing for the use of a validated theoretical model, namely, a more precise computational analysis [11,12]. As a result, the present study aimed to evaluate the biomechanical behavior of PEEK abutments with different heights on single titanium implants. The null hypothesis tested was that all methodologies would present similar results.

## 2. Materials and Methods

### 2.1. Experimental Desing

Forty implants (4 × 10 mm) of morse taper (B&B Dental, San Pietro in Casale, Italy) made of commercially pure titanium were used. Herein, 20 implants received the 4.5 × 4.0 mm PEEK short abutment (SA) and 20 implants received the 4.5 × 5.5 mm PEEK long abutment (LA). These pillars feature a 6° conical design and internal friction fit. The abutments were installed and fitted using Panavia V5 dual-cure resin cement (Kuraray Noritake Dental Inc., Okayama, Japan). The hemispherical shape of the device allowed the loading of the load to be applied at a single point in the central area of the column in all samples, thus avoiding uneven concentration of forces as well as the premature strain of the samples.

The specimens were made using PVC tubes with a diameter of ¾ inch, where the implants were stabilized by a polyurethane resin (F160, Axson Technologies, Saint-Ouen-I’Aumône, France). In accordance with the ISO for fatigue of dental implants ISO 14801:2016, 3 mm of the implants will remain exposed above the resin simulating an unfavorable clinical condition [13]. The tests were performed by a single trained operator and the samples were randomly numbered before the tests were performed within their groups.

### 2.2. Surface Topography (SEM/FEG)

Representative specimens of the dental implants were analyzed for their surface morphology by scanning electron microscopy (SEM, Inspekt S50, FEI Company, Brno, Czech Republic).

### 2.3. Energy Dispersive X-ray (EDX)

Specimens were also subjected to an energy-dispersive spectroscopy analysis to evaluate the chemical composition of the surface (Inspekt S50, FEI Company, Brno, Germany) associated with Espirit 1.9 software (Bruker, Berlin, Germany).

### 2.4. X-ray Diffraction Analysis (XRD)

A high-resolution X-ray diffractometer (GIXRD; Philips X’pert PRO MRD, Almelo, The Netherlands) was used to identify the composition of the materials. The identification of the alloy composition was given after comparing the experimental spectra with standard diffraction spectra from the JCPDS (Joint Committee on Powder Diffraction Standards) and ICSD (Inorganic Crystal Structure) database, at a pre-fatigue test in all the groups.

XRD standard incidence scan was performed on the Bragg θ-2θ geometry, equipped with a graphite monochromator and Cu Kα radiation (λ = 1.5406 Å), operating at a voltage of 40 kV and an emission current of 40 mA. Data were obtained in 2θ ranging from 20° to 70° at a scan rate of 0.2°/min and a step size of 0.02°. Quantitative phase analysis was performed using the Rietveld refinement method in X’Pert HighScore software (Malvern PANalytical Ltd., Westborough, MA, USA), which estimates the weight fraction (%) of each phase based on the relative peak intensity.

### 2.5. Maximum Fracture Load

To determine the fatigue test that was used, two specimens from each group were submitted to the single load fracture test. For this, the samples were fixed on a base with an angle of 30° about the ground (ISO 14801:2007) and were submitted to a compressive load of 0.5 mm/min in a universal testing machine (Emic DL-1000, Emic, São José dos Pinhais, Brazil).

### 2.6. Fatigue Analysis

For the fatigue test, the samples were positioned on a 30° angle base of the mechanical fatigue simulator (RE 11.000 Plus, Erios; São Paulo, Brazil) and received 2,000,000 cycles at a frequency of 2 Hz with 1.6 mm diameter stainless steel pistons, according to the parameters described in the standard ISO 14801:2016. The test was performed with the samples embedded in distilled water at 37 °C. Fatigue strength analysis was performed in sequence using the step-stress test [14]. The samples were tested in a mechanical fatigue machine (Biocycle, Biopdi, São Carlos, Brazil), in the presence of distilled water with the same device as the monotonic test, inclined at 30°, with a frequency of 10 Hz [15]. Load profiles were analyzed starting at 100 N with the load increasing at each following profile, at intervals of 10,000 cycles. The number of cycles and the load at which the samples fractured during the fatigue test was analyzed by the reliability software SPSS statistics (IBM, Chicago, IL, USA) using the survival analysis function, Kaplan-Meier, and Mantel-Cox tests (log-rank) (*p* < 0.05).

### 2.7. Finite Element Analysis (FEA)

Using CAD software (Rhinoceros 5.0, McNeel Europe™, Barcelona, Spain), all structures were modeled according to the specifications and geometry of each material and the set gave rise to the final model for each group [16]. Therefore, a three-dimensional (3D) structure was modeled to represent a cylinder-shaped section (10 × 20 mm). Based on the therapeutic possibilities for the same clinical indication, a single system with the same characteristics and mechanical properties of the structures was performed. However, a slight difference could be noticed in the height of the column [16,17]. The models were divided into the respective study groups: Short abutment (SA) 4.5 × 4.0 mm (control) and long abutment (LA) 4.5 × 5.5 mm (experimental) (B&B Dental, San Pietro in Casale, BO, Italy).

**Figure 1:**
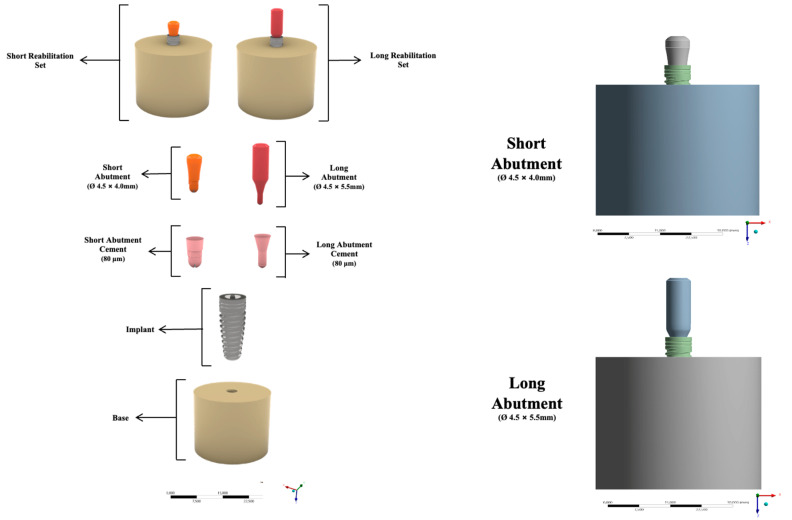
Illustrative of 3D models following the clinical sequence of an implant-supported prosthesis using the short abutment and long abutment implant systems.

Then, the specimen section of the specimen was implanted (Morse taper—4 × 10 mm, Bone Level, B&B Dental, San Pietro in Casale, BO, Italy) according to the system of each group. To simulate a condition closer to the clinical condition already reported in the literature [17], the entire implant and abutment set was positioned on the base as shown in Figure 1.

**Figure 2:**
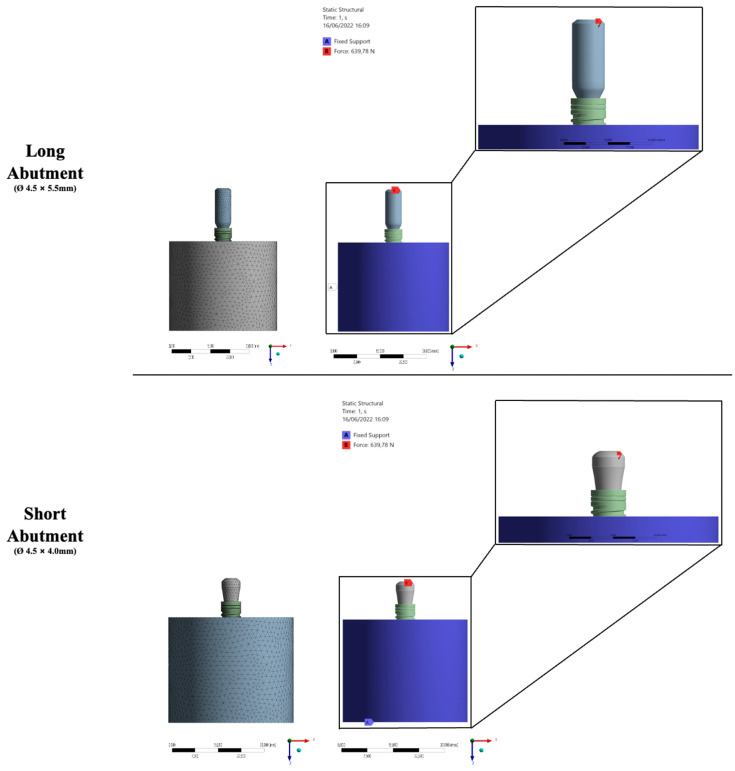
FEA details—Mesh, boundary conditions, loads, and connections.

Then, the solids were exported in STEP (STandard for the Exchange of Product model data) format for software analysis (ANSYS 17.2, ANSYS Inc., Houston, TX, USA). The outer surface of the specimen section was fixed in all directions, applying an oblique load of 100 and 200 N was applied to the central surface of the prosthetic abutment, specifically at the entrance of the prosthetic screw. A mesh was created after the 10% convergence test [16,18] corresponding to 952,108 nodes and 709,276 tetrahedral elements for the evaluated models (Figure 2). All materials were considered isotropic, linear, elastic, and homogeneous. Between the implant and the bone, the contact was used to simulate complete osseointegration [18], and the other contacts were considered glued.

The mechanical properties of the 3D model structures were defined based on the literature (Table 1). The results of the stress distribution according to the von Mises criteria were exhibited using visual plots with a scale in megapascals (MPa) for implants and structures. The microstrain criteria were used to investigate the bone behavior and its maximum values are shown in Table 2 [19,20].

Stress and strain results that present values with a difference of less than 10% may be located in the convergence range of the analysis software, making it impossible to assume a significant difference. Consequently, the results that present a difference in peak values greater than 10% will be defined as significant. The results of each structure of both groups were compared qualitatively and quantitatively. Microstrain, von Mises stress, and maximum principal stress were adopted as failure criteria. Statistical tests were performed in SPSS statistical software version 21 (SPSS Inc., Chicago, USA). The level of significance established for the Kaplan-Meier and Mantel-Cox tests (log-rank) was 5%, which established a 95% confidence interval for the presented results, and the power of a statistical test was 80%.

## 3. Results

### 3.1. Surface Topography (SEM/FEG)

**Figure 3:**
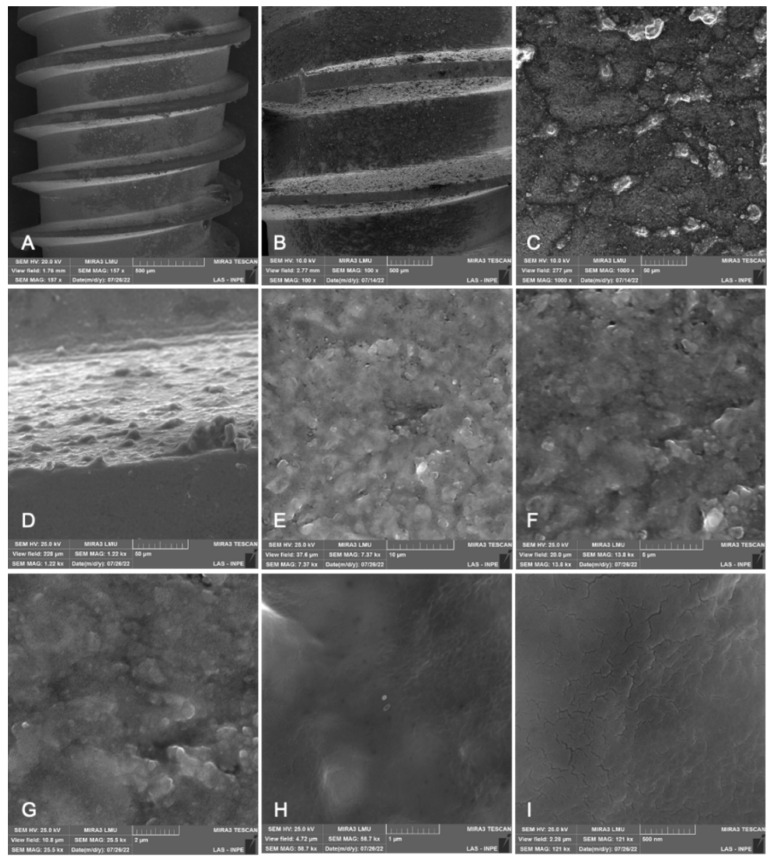
Micrographs of representative samples of titanium implants. SEM images of the surface at different magnifications: (**A**) 57×; (**B**) 100×; (**C**) 1000×; (**D**) 1220×; (**E**) 7370×; (**F**) 13,800×; (**G**) 25,500×; (**H**) 58,700×; (**I**) 121,000×.

SEM images demonstrated representative samples from the experimental specimen. No topographical differences were observed on the surfaces of the materials under study. The implant presented more homogeneous surfaces in the magnifications of 20 and 100×, while in the magnifications of 50,000 and 100,000× it is possible to observe irregular surfaces, with the presence of porosities (Figure 3).

### 3.2. Energy Dispersive X-ray (EDX)

**Figure 4:**
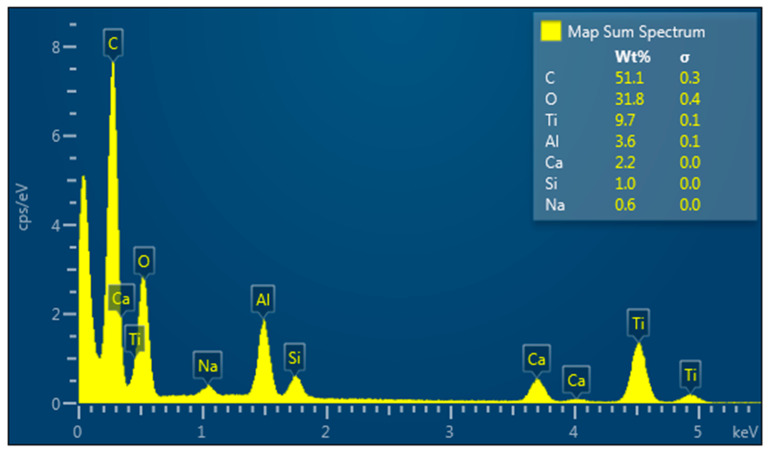
Specimen composition was characterized through energy dispersive X-ray (EDX) analysis.

Energy dispersive X-ray (EDX) analysis of the implant body shows a strong signal in the titanium oxide and chloride region. This affirms the generation of grade IV titanium particles with a high percentage as described in Figure 4. The other peaks are due to some impurities in the sample, which may come from the aluminum chloride sample. A relatively small signal is attributed to the formation of sodium and silica at about 0.9 keV. This can be attributed to the amount of sodium subjected to air that reacts with active carbon after synthesis and transfers to the analysis. The same compounds were observed as surface treatment of the implant; however, in different concentrations of chemical elements. Moreover, the EDS analysis allowed for measuring the percentage of each chemical element (%at) according to the weight of each element in the analysis (%wt).

### 3.3. X-ray Diffraction Analysis (XRD)

**Figure 5:**
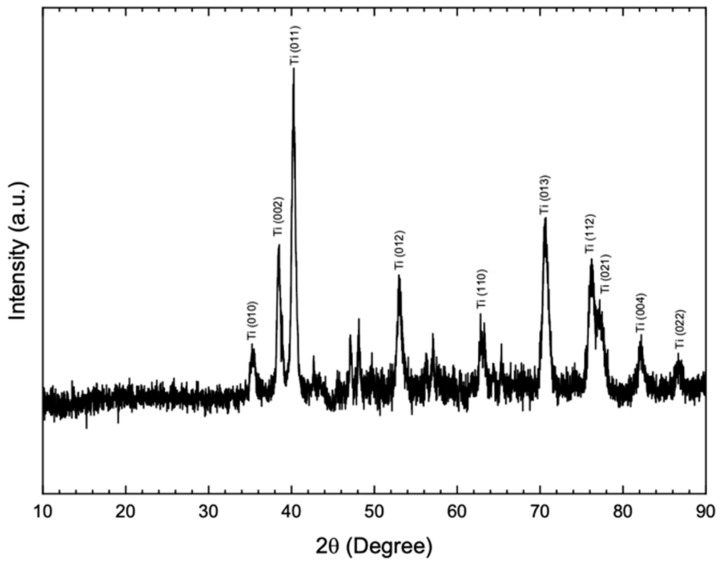
X-ray diffractograms corresponding to the titanium implant before and after the analysis of survival in fatigue by artificial aging.

The X-ray diffractograms shown in Figure 5, corresponding to the titanium implant, (A) describe that its surface is mostly composed of titanium oxide. Since it has two peaks corresponding to the alpha (α) and beta (β) phases, this is a characteristic of this type of titanium alloy.

### 3.4. Maximum Fracture Load

The results of the analysis of the maximum load for fracture are described in Table 2. No statistically significant differences were found (*p* = 0.189) for the analyzed groups. The macrogeometry and height of the prosthetic component were not able to influence the mechanical strength of the set (Table 2).

### 3.5. Fatigue Analysis

**Figure 6:**
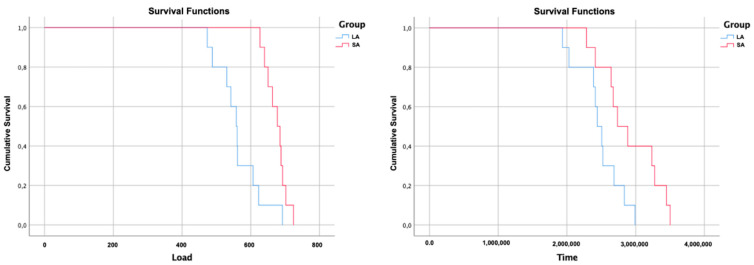
Survival graph of groups as a function of load (N) and time (cycles). Legend: Survival profile between SA (short abutment) and LA (long abutment) groups. As a function of load and time per number of cycles, until failure of the implant/abutment set.

Due to the absence of failure of any sample at the end of 2,000,000 cycles at a frequency of 2 Hz with a load of 200 N (thermomechanical aging cycling), the step-stress test was also performed. Therefore, the means and confidence intervals of fracture resistance and cycles for fracture were obtained in the Kaplan-Meier and Mantel-Cox tests (log-rank, 95%), presented in Table 3. The study groups (SA and LA) were statistically different regarding the number of cycles required for the fracture of the implant/abutment set (*p* = 0.022), and regarding fracture resistance, both groups presented statistically significant values (*p* = 0.001) (Figure 6).

### 3.6. Finite Element Analysis (FEA)

**Figure 7:**
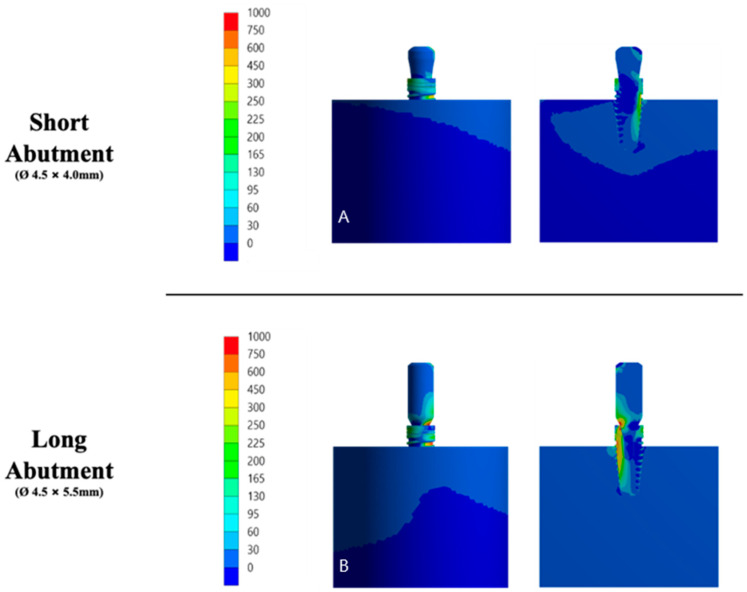
Illustrative of the 3D modeling qualitative analysis of microstrain (**left**) and displacement (**right**) criteria on the structures of each group. (**A**) Short abutment. (**B**) Long abutment.

**Figure 8:**
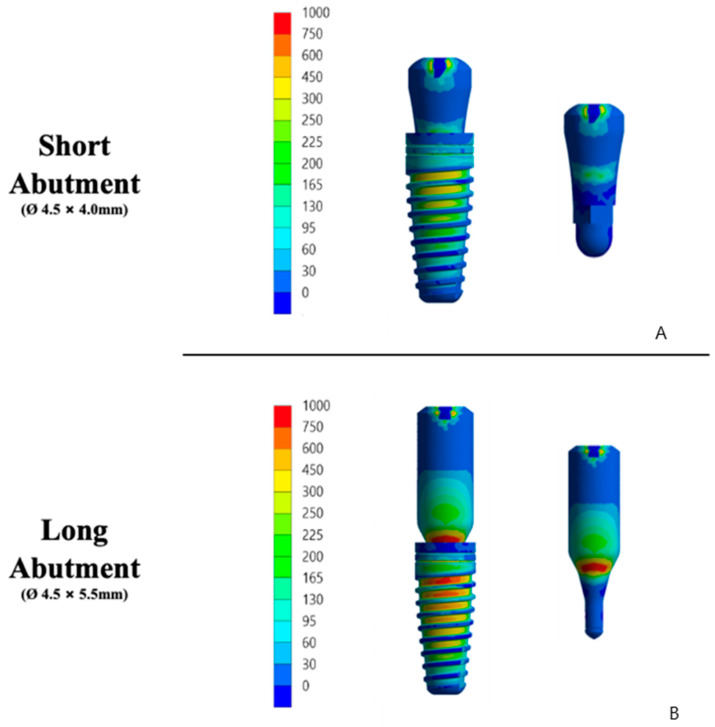
Illustrative of the 3D modeling of the qualitative analysis of maximum principal stress and von Mises (Mpa) criteria on the implant/abutment (**left**) and abutment (**right**) of each group. (**A**) Short abutment. (**B**) Long abutment.

In an extensive qualitative analysis, the displacement of the implant-abutment assembly can be verified in Figure 7. The mechanical responses were calculated according to the failure criteria of each structure. Based on previous studies, bone tissue results were analyzed on the strain [21,22,23]. Observing the strain distribution represented in Figure 8, it is possible to observe that there is a similar response pattern among the models for the strain generated in the cortical bone; however, the long abutment model has a greater magnitude of cervical tension than the short abutment model.

This behavior can be described by the tensile and von Mises stress map in Figure 8. In ductile solids, such as titanium implants and abutments, the stress results followed the von Mises criteria that help in showing the regions of onset of fracture in metals. However, for a comparative analysis between the models, the results were obtained by the von Mises stress, and the model with a long abutment showed higher stress concentration. This highlights the tensile stress region, which is the failure criterion for metals. Therefore, both criteria present similar stress maps, but with different magnitude values, as plotted in Table 4. As a result, the long column concentrates more stresses in its structure, reducing the energy required for its displacement (Figure 8).

## 4. Discussion

The objective of this study was to evaluate the in vitro and silico biomechanical behavior of abutments with different PEEK prosthetic collar heights cemented directly into the internal connection of morse-taper implants as a non-metallic alternative to titanium abutments coupled to the internal structure through the fitting. Both PEEK abutments showed significantly lower mechanical resistance to fatigue than the titanium ones, but for the group with the long neck, there was a greater compromise in the bone crest of the ridge, consecutively a greater possibility of microleakage in the region of the abutment-implant interface. Therefore, the heights of PEEK abutments lead to different behaviors, and the null hypothesis was rejected.

Due to a need to develop abutments with alternative alloys to titanium for patients who have allergies to metals, new materials made from polymers are produced in the market. These materials have good resistance to fracture, allowing for the distribution of tension forces and traction to the surrounding peri-implant tissues during mastication [22,23,24]. In addition, PEEK abutments are interesting alternative therapies as they prevent screw loosening and misfit in the internal connection of the implant, in cases where they are rehabilitated with screw connections. Therefore, these conditions that allow for intimate mechanical imbrication, result in preventing microleakage and bacterial colonization in the interface region [23,24].

PEEK has limitations as an alternative to titanium, even in situations where the material is reinforced with other compounds, thus improving biomechanics and providing a more resilient definitive implant abutment [22,25]. However, all PEEK abutments have clinically relevant disadvantages, including their color which after the aging process becomes black, as well as greater accumulation of microorganisms, resulting in greater inflammation of the peri-implant tissue due to significant roughness in the abutment [15,22,23,26].

In the present study, the high presence of carbon oxide on the surface of the implant, both internally and externally, can be observed, which may result in a greater capacity for the mechanical imbrication of the internal connection [27]. Therefore, higher surface energy can be observed, before an imminent difficulty in the rupture between the structures joined through resin cement, which is the case of our study. In addition to the fact that both the implant and the abutment have grooves throughout the body, this favors a more precise deposition and adaptation of the set when installed in function. However, this surface treatment present in the set does not result in greater strength of the material. Since the average values of fracture resistance were 563.57 N for the long and 674.99 N for the short abutments, values between the minimum (95 N) and maximum (750 N) masticatory forces were reported. In addition, a fatigue load test was performed at 200 N for 2 million cycles (equivalent to 10 years of function), and there was no failure of both PEEK abutment, but it did cause significant vertical strain in the long PEEK abutment. When the load was increased to 780 N, the PEEK abutments did not exceed 350,183 cycles (equivalent to approximately 20–24 months of occlusal function) [28].

Furthermore, both PEEK abutments acted as the sacrificial material, absorbing all plastic strain and breaking before implantation. However, a slight difference can be observed in both components, where the long abutment group concentrated greater plastic strain in the internal connection, compromising the viability of the implant after overload. Specifically, overloads using the long abutments caused permanent strain on the internal connection [29,30,31].

This higher concentration observed in the internal connection can result in a misfit at the implant-abutment interface that facilitates bacterial microleakage, in more advanced cases leading to fracture of the component inside the implant [32,33]. PEEK abutments exhibited large vertical displacement and plastic strain during the dynamic fatigue test, possibly promoting microgaps at the implant-abutment interface [27,29,34,35]. Studies demonstrate that the displacement of PEEK pillars is not only caused by dynamic fatigue, but also by the increase in material temperature [29,30,31,36,37]. Since the increase in loading frequency results in an increase in PEEK temperature with consequent plastic strain, this produces premature loosening of the rehabilitation set and microleakage. Microleakage is one of the main phenomena caused by the lack of fit at the implant-abutment interface. Consecutively, a misfit between the abutment and implant creates a site for a bacterial colonization that can trigger peri-implant inflammatory processes, followed by implant loss in more advanced cases [36,37,38,39,40].

In the present study, the incidence of stress in the long abutments was significantly higher than in the short abutments. This difference is probably related to a higher incidence of failure of columns made entirely from PEEK since they present high plastic strain during dynamic mechanical tests [14,21,34]. In this sense, an ideal connection between the abutment and the implant should minimize the space between the components when subjected to occlusal forces. The present finding suggests that a gap between the abutment and the implant may appear during functional use. However, it can be inferred that abutments made entirely from PEEK, joined to the internal connection of the implant through a cementation process, do not seem to be an interesting alternative in situations of oral rehabilitation.

The mechanical fatigue test is one of the most used to investigate the performance of restorative materials. The specificity of these investigations makes it possible to understand clinical manifestations; however, they limit the extrapolation of their results. In this sense, the finite element method can present alternative results in a clear and non-destructive way [31]. Therefore, the present study investigated different heights of PEEK abutments using different methodologies. The use of different methods for the same investigation with results that corroborate each other allows for reaching results that are closer to reality. There are a few noteworthy limitations to this study including the simplification of the PEEK abutment, the absence of an anatomical crown, in addition to the homogeneity of the structures of the 3D models. Moreover, future studies should analyze the misfit between a dental implant and a PEEK prosthesis made with different manufacturing processes and anatomic designs, combining dynamic mechanical testing and an artificial mouth. More studies are needed to validate PEEK as an alternative material for the fabrication of temporary abutments in different clinical settings.

## 5. Conclusions

Based on the results of this in vitro and in silico study, the following conclusions were drawn:

1. PEEK abutments can withstand moderate forces and should be indicated with caution for implant-supported rehabilitation;
2. Limitations of PEEK abutments include a large vertical displacement and plastic strain at the abutment-implant interface;
3. PEEK abutments may be suitable as temporary rehabilitation, especially in the anterior region and for patients without parafunction, but joint failure must be considered.

## Figures and Tables

**Table 1:** Mechanical properties of the materials.

Material	Young’s Modulus (GPa)	Poisson Ratio
Titanium [14]	110	0.30
PEEK [18]	3.7	0.40
Co-Cr [17]	218	0.30
Zirconia [18]	220	0.30
Carbon-reinforced polymer [19]	42.7	0.30
Polyurethane [20]	3.6	0.30

**Table 2:** Mean value ± standard deviation (SD) of the data obtained by resistance in the analysis of maximum load for fracture for the titanium implant group with long PEEK abutment and group.

Groups	Mean Value (kgf)	SD	CI 95%
Short Abutment	64.1 ^a^	±3.57	(67.67–60.53)
Long Abutment	56.5 ^a^	±6.14	(62.64–50.36)

Legend: Different letters indicate statistically significant in the column.

**Table 3:** Mean value ± standard deviation (SD) of the data obtained by resistance in the analysis of maximum load for fracture for the titanium implant group with long PEEK abutment and group.

Fatigue Failure Load				
Group	Mean Value (kgf)	SD	CI 95%—Minimum	CI 95%—Maximum
SA	674.99 ^a^	±9.44	(656.48)	(693.51)
LA	563.57 ^b^	±20.36	(523.67)	(603.48)
**Number of Cycles to Fatigue Failure**				
Group	Mean Value	SD	CI 95%—Minimum	CI 95%—Maximum
SA	290,964.41 ^a^	±13,651.93	(264,206.61)	(317,722.19)
LA	247,494.71 ^b^	±10,249.97	(227,404.74)	(267,584.65)

Legend: Different letters indicate a statistically significant difference in the column.

**Table 4:** Quantitative analysis of von Mises stress results in stress peaks (MPa) in the structures and bone microstrains (με) of each group.

Analysis Criterion	Group	
	LA	SA
Displacement (mm)	0.42	0.56
Microstrain (µm/µm)	0.0780	0.0798
Maximum Principal Stress (MPa)	280.76	250.11
von Mises Stress (MPa)	266.69	278.39

## Data Availability

Data are available upon request.
